# Decomposition and comparative analysis of depressive symptoms between older adults living alone and with others in China

**DOI:** 10.3389/fpubh.2023.1265834

**Published:** 2023-09-22

**Authors:** Chaoqun Hu, Zhixin Dai, Hao Liu, Suiyi Liu, Maolin Du, Tongtong Liu, Lei Yuan

**Affiliations:** ^1^Faculty of Military Health Service, Department of Health Management, Naval Medical University, Shanghai, China; ^2^Department of Medical Engineering, Shanghai Eastern Hepatobiliary Surgery Hospital, Naval Medical University, Shanghai, China; ^3^Department of Office, Naval Medical University, Shanghai, China

**Keywords:** depressive symptoms, older adults, living status, China, Fairlie decomposition

## Abstract

**Objective:**

This research dealt with investigating and measuring the contribution of the factors that impact depression in older adults living alone vs. those living with others (hereafter referred to as “not alone”) in China.

**Design:**

This investigation adopts a cross-sectional research design. The dataset employed for this study comprises data from 2018 the Chinese Longitudinal Health Longevity Survey (CLHLS).

**Setting:**

The research involved data sourced from China, specifically from 23 of its provinces. From the 8th CLHLS, 12,197 older adults were selected who met the study requirements.

**Measures:**

Binary logistic regression models were established to delve into the primary factors impacting the depressive symptoms of the individuals. Furthermore, Fairlie models were employed to assess these factors between older adults living alone and those not living alone. This approach facilitated an in-depth analysis of their respective contributions.

**Results:**

It was observed that the demographic of Chinese older adults exhibited depressive symptoms at a rate of 11.92%. Older adults who resided alone (15.76%) exhibited a higher prevalence of depressive symptoms in comparison to their counterparts living in not-alone settings (11.15%). Employing Fairlie decomposition analysis, it was determined that this observed disparity in depressive symptoms, amounting to 55.33% of the overall difference, could be primarily attributed to distinct factors. This encompassed variance in marital status (20.55%), years of school (4.63%), self-reported local income status (7.25%), self-reported sleep status (17.56%), and self-reported health status (4.24%).

**Conclusion:**

The resulting data indicated that depressive symptoms exhibited an elevated prevalence in older adults living alone than in those living not alone. This discrepancy was predominantly attributed to variance in socioeconomic marital status, years of school, self-reported local income status, self-reported sleep status, and self-reported health status by living alone vs. not alone. Mitigating these influential factors could help develop targeted and meticulous intervention strategies, precisely tailored to improve the mental well-being of older adults at high risk.

## 1. Introduction

Depression is a mood disorder that causes a persistent feeling of sadness and loss of interest. The common features of all the depressive disorders are sadness, emptiness, or irritable mood, accompanied by somatic and cognitive changes that significantly affect the individual’s capacity to function (1, 2). Because of false perceptions, nearly 60% of people with depression do not seek medical help and depression exerts a negative influence on the quality of life experienced by older adults (2). It primarily impacts older adults who are afflicted with chronic medical conditions and cognitive impairment. This condition precipitates personal distress, familial discord, and functional impairment, exacerbating the prognosis of various illnesses like diabetes, autoimmune disorders like rheumatoid arthritis, lupus, and cardiovascular diseases like coronary heart disease, hypertension (HTN), obesity, physiological aging, cancer, poor hearing, and poor health and heightening mortality rates (3, 4). Furthermore, it catalyzes suicidal tendencies, culminating in adverse health ramifications that result in a considerable burden on both families and society at large (5, 6).

Due to the accelerated process of global aging, the global population of older adults surpassed the 1 billion mark in 2021, constituting approximately 13.5% of the global populace. As projected by the World Health Organization, by the year 2030, an estimated one in six individuals will be age 60 years or older, underscoring the escalating significance of this demographic shift (7). As the country that long had the largest population worldwide, but is now the second largest and shrinking, China is facing an especially serious problem of an aging population. As per the findings of the seventh National Census conducted in 2021, the cohort of individuals aged 60 years and above within China has reached a substantial count of 260 million, comprising 18.7% of the entire population. Within this segment, individuals aged 65 years and above constitute 190 million individuals, equivalent to 13.5% of the total population (8). Projections indicate that the proportion of older adults aged 65 and above in the overall population will reach about 26.9% by the year 2050 (9).

Additionally, the implementation of the family planning policy in China has led to drastic declines in fertility, distinct from the demographic changes even in other countries with fertility declines over the past three decades. The 4 (older adults)–2 (a young or middle-aged couple)–1 (a child) family structure has become mainstream (10). Simultaneously, within contemporary society, adult children increasingly seek autonomy and their own personal “free space.” This trend has contributed to the erosion of the traditional family model characterized by multi-generational cohabitation involving three or even four generations. The functioning of family older-adult care is weakening and shifting to social eldercare services. In China, where cultural norms emphasize family structure and collective values, the family has traditionally served as a vital support system for older adults. As their spouses, cohabitants, or important friends die, the issue of older adults living alone has become a social concern and has garnered significant attention (11).

Are there differences in depressive symptoms between older adults living alone and not alone? Numerous investigations have established a robust correlation between loneliness in old age and the occurrence of depression (11, 12). A comprehensive review systematically elaborates on living alone may predispose individuals to an elevated susceptibility to psychiatric conditions such as depression, alcohol abuse, sleep disorders, personality disorders, and Alzheimer’s disease (4). One approach to preventing depression in older adults living alone is to identify factors distinguished from older adults living not alone. The factors may include sex, self-perceived financial status, marital status, educational level, living status, self-reported health status (SRH), quality of social relationships, smoking, and alcohol consumption (13–18). Therefore, we must explore the importance and contribution level of each factor between living alone and not alone, which will help us develop targeted measures to reduce depression in older adults living alone.

So further assessment of the elements that result in heightened depression in older adults living alone in comparison with those living with others (hereafter referred to as “not alone”) must be executed. Furthermore, this research attempted to establish a foundation for the formulation of effective policies aimed at managing the levels of depressive symptoms experienced by older adults. Therefore, the variance between older adults (aged 65+) living alone and those cohabitating was examined in China. To achieve this, the Chinese Longitudinal Health Longevity Survey (CLHLS) dataset was utilized. Initially, the study delved into the degree to which sociodemographic characteristics, personal lifestyle choices, and health statuses accounted for the variations in depressive symptoms among Chinese older adults living alone and not alone.

## 2. Methods

### 2.1. Data sources

**Figure 1:**
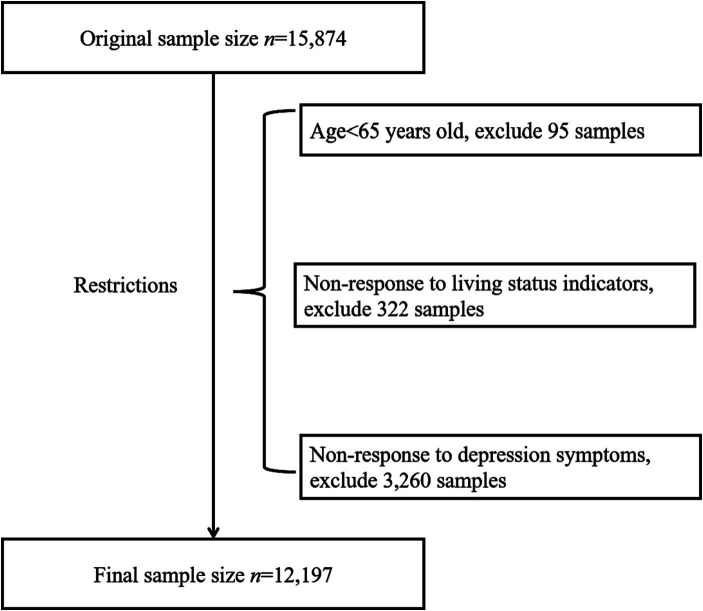
Flowchart of participants.

The data were retrieved from the 8th CLHLS (PKU Center for Healthy Aging and Development, 2020). Detailed information regarding the sources and design of the datasets can be accessed at https://opendata.pku.edu.cn/dataset.xhtml?persistentId=doi:10.18170/DVN/ WBO7LK (Accessed July 22, 2023). The CLHLS was executed by the Center for Healthy Ageing and Development Studies/National Development Research Institute of Peking University and was subjected to approval by the Ethics Committee of Peking University (No. IRB00001052-13074). The survey comprehensively spanned 23 of the provinces, encompassing approximately two-thirds of the geographical expanse of China. The focal demographic comprised individuals aged 65 years and above. The methodology involved two distinct questionnaires: one aimed at respondents who were alive and another tailored for family members of deceased older adults. The 8th iteration of the CLHLS took place from 2017 to 2018, involving interviews with a total of 15,874 older adults. We selected the participants who completed the full 10-question version of the Center for Epidemiology Studies Depression (CES-D) questionnaire were selected. The exclusion criteria encompassed individuals below the age of 65, as well as those who had not responded to measurements relating to depressive symptoms, demographic and sociological characteristics, personal lifestyle choices, or health status indicators. Finally, 12,197 respondents were selected for this study (Figure 1). Ultimately, including 2029 and 10,148 older adults living alone and not alone, respectively. The process utilized for the exclusion of non-relevant individuals is depicted in Figure 1.

### 2.2. Depressive symptoms

The CES-D has been widely used as a practical depression screening tool by previous studies targeting older adults in China (19–21). In the 8th CLHLS, the CES-D-10 scale comprises 10 specific items such as “I was bothered by things that usually do not bother me,” “I did not feel like eating; my appetite was poor,” to comprehensive evaluation of the responders’ depression status. The response options for each item range from 0 (*none of the time* or *rarely*) to 3 (*most* or *all the time*) on the questionnaire. Higher scores represent more severe depressive symptom. The questionnaire was centered on assessing depressive symptoms within the context of the past week. In the same way that multiple previous studies defined a score of 10 as a threshold score, participants with a score greater than or equal to 10 were defined as experiencing depressive symptoms (20, 22). Although scoring above a 10 does not directly correlate with a formal diagnosis, it does indicate a need for direct clinical assessment (22).

### 2.3. Group variables

Respondents were classified as alone or not alone based on whether they cohabited with family or spouse during the survey duration.

### 2.4. Covariates

The reliability of the acquired data was enhanced further by accounting for multiple potential confounding factors. To acquire comprehensive factors spanning sociological attributes, demographic characteristics, sociological characteristics, personal lifestyle and health status were drawn from existing studies on depressive symptoms.

#### 2.4.1. Demographic characteristics

Age was classified as <70 years, 70–79 years, 80–89 years, 90–99 years, or > =100 years. The residence mainly included city, town, or rural. BMI was calculated by dividing weight (kg) by the square of height (m) and was divided into four categories: <18.5, 18.5–23.9, 24.0–27.9, and > =28.0. Education level was classified according to time in school as 0 years, 1–6 years, and ≥ 7 years.

#### 2.4.2. Sociological characteristics

Marital status included married and living with spouse, widowed, and others (including married but not living with a spouse, divorced, and never married). Self-reported local income status was divided into three categories: poor, average, and rich.

#### 2.4.3. Personal lifestyle

Personal lifestyle included smoking, drinking, exercise, and self-reported sleep status. Smoking, drinking, and exercise were categorized as “Yes” or “No” based on the responses to specific questions. Specifically, questions like “Do you currently drink alcohol,” “Do you presently smoke?” and “Do you do regular exercises?” Self-reported sleep status was based on the question “What do you think of your recent sleep situation?” and classified as Poor, Average, and Rich.

#### 2.4.4. Health status

Health status included SRH, hypertension, heart disease, diabetes, and stroke. The assessment of SRH was contingent on the answer to the question “How do you rate your current health?” This was followed by the categorization of the resulting data as average (so-so), good (good or very good), and poor (bad or very bad). We carefully asked each person about the four types of chronic diseases—hypertension, heart disease, diabetes, and stroke—and each item was categorized as “Yes” or “No.”

### 2.5. Statistical analyses

Demographic and sociological characteristics, personal lifestyle choices, and health statuses were investigated using descriptive statistics. The distribution patterns of depressive symptoms among older adults living alone and those living in not-alone arrangements were analyzed using the chi-squared test. Subsequently, the binary logistic regression model was applied to assess the primary influencers of depressive symptoms within the contexts of both living conditions. These statistical analyses were executed utilizing SPSS 21.0 software. To further investigate the dynamics influencing and contributing to the divergence in depressive symptoms between older adults living alone and those cohabitating, the Fairlie model was employed. However, for the missing data of covariates, we used the multiple-imputation mothed to simulate, with each variable supplemented 10 times.

The software Stata MP16.0 was utilized for the assessment of data. *The level of statistical significance was defined as 0.05*.

#### 2.5.1. Fairlie decomposition analysis

Given that the dependent variable takes on two distinct values (a dichotomous variable), the Fairlie nonlinear decomposition approach was applied. This methodology allowed the decomposition of the variations in depressive symptoms by attributing them to different contributing factors. More details are provided in our previous studies (23, 24).

## 3. Results

### 3.1. Respondents’ general data

In total, this research involved 12,197 individuals. The outcomes of the descriptive statistical analyses for the older adults living alone and not alone in China are illustrated in Table 1. The resulting data indicated that 11.92% of these aged people had experienced depressive symptoms, while 88.08% had none. An elevated proportion of older adults living alone (15.76%) had experienced depressive symptoms in contrast to those living not alone (11.15%) (*p* < 0.001). The resulting data of a chi-squared test depicted variance in the distribution of the 10 covariates across the two living conditions. These encompassed: age, residence, sex, BMI, marital status, education level, self-reported local income status, self-reported sleep status, diabetes, and stroke.

**Table 1:** Basic information of participants.

Variables	Alone [*n* (%)]	Not alone [*n* (%)]	*c* ^2^	*P*
CES-D 10			34.636	<0.001
<10	1,726 (84.24)	9,017 (88.85)		
^3^10	323 (15.76)	1,131 (11.15)		
Age (years)			208.752	<0.001
<70	126 (6.15)	1,302 (12.83)		
70–79	512 (24.99)	2,945 (29.02)		
80–89	781 (38.12)	2,618 (25.80)		
90–99	469 (22.89)	2,002 (19.73)		
^3^100	161 (7.86)	1,281 (12.62)		
Residence			37.464	<0.001
City	376 (18.35)	2,493 (24.57)		
Town	704 (34.36)	3,313 (32.65)		
Rural	969 (47.29)	4,342 (42.79)		
Sex			58.160	<0.001
Female	1,267 (61.84)	5,263 (51.86)		
Male	782 (38.16)	4,885 (48.14)		
BMI (kg/m^2^)			8.383	0.039
18.5–23.9	1,048 (51.15)	4,990 (49.17)		
<18.5	306 (14.93)	1,414 (13.93)		
24.0–27.9	451 (22.01)	2,490 (24.54)		
^3^28.0	186 (9.08)	828 (8.16)		
Missing	58 (2.83)	426 (4.20)		
Marital status				
Married and living with a spouse	114 (5.56)	5,288 (52.11)		<0.001
Widowed	1,771 (86.43)	4,530 (44.64)		
Other	145 (7.08)	227 (2.24)		
Missing	19 (0.93)	103 (1.01)		
education level			78.619	<0.001
0	913 (44.56)	3,741 (36.86)		
1–6	559 (27.28)	3,048 (30.04)		
^3^7	256 (12.49)	1,995 (19.66)		
Missing	321 (15.67)	1,364 (13.44)		
Self-reported local income status			70.116	<0.001
Poor	277 (13.52)	906 (8.93)		
General	1,426 (69.59)	6,971 (68.69)		
Rich	315 (15.37)	2,197 (21.65)		
Missing	31 (1.51)	74 (0.73)		
Smoking			0.175	0.676
No	1,705 (83.21)	8,425 (83.02)		
Yes	321 (15.67)	1,631 (16.07)		
Missing	23 (1.12)	92 (0.91)		
Drinking			0.015	0.901
No	1,710 (83.46)	8,451 (83.28)		
Yes	309 (15.08)	1,540 (15.18)		
Missing	30 (1.46)	157 (1.55)		
Exercise			1.850	0.174
No	1,343 (65.54)	6,528 (64.33)		
Yes	669 (32.65)	3,489 (34.38)		
Missing	37 (1.81)	131 (1.29)		
Self-reported sleep status			24.893	<0.001
General	685 (33.43)	3,216 (31.69)		
Bad	367 (17.91)	1,458 (14.37)		
Good	997 (48.66)	5,474 (53.94)		
SRH			3.787	0.151
General	821 (40.07)	3,871 (38.15)		
Bad	277 (13.52)	1,328 (13.09)		
Good	951 (46.41)	4,946 (48.74)		
Missing	0	3 (0.03)		
Hypertension			0.802	0.371
No	1,029 (50.22)	5,210 (51.34)		
Yes	882 (43.05)	4,269 (42.07)		
Missing	138 (6.73)	669 (6.59)		
Heart disease			0.004	0.948
No	1,485 (72.47)	7,428 (73.20)		
Yes	343 (16.74)	1,723 (16.98)		
Missing	221 (10.79)	997 (9.82)		
Diabetes			4.909	0.027
No	1,644 (80.23)	8,069 (79.51)		
Yes	177 (8.64)	1,050 (10.35)		
Missing	228 (11.13)	1,029 (10.14)		
Stroke			8.195	0.004
No	1,642 (80.14)	7,998 (78.81)		
Yes	175 (8.54)	1,094 (10.78)		
Missing	232 (11.32)	1,056 (10.41)		

### 3.2. Comparison of variable distribution across depressive symptoms

The distribution of covariates was assessed across older adults living alone and those living with someone else with different depressive symptoms. The data are depicted in Table 2. The resulting data demonstrated that certain covariates exhibited dissimilar distribution patterns among older adults with and without depressive symptoms. These divergent characteristics were evident in the variables of sex, self-reported sleep status, self-reported health status, diabetes, and stroke.

**Table 2:** Alone vs. not alone older adults differential in depression symptoms and non-depressive symptoms by selected background characteristics.

Variables	Non–depression symptoms			Depression symptoms		
	Alone [*n* (%)]	Not alone [*n* (%)]	*P*	Alone [*n* (%)]	Not alone [*n* (%)]	*P*
Age (years)			<0.001			<0.001
<70	103 (5.97)	1,181 (13.10)		23 (7.12)	121 (10.70)	
70–79	443 (25.67)	2,637 (29.24)		69 (21.36)	308 (27.23)	
80–89	646 (37.43)	2,320 (25.73)		135 (41.80)	298 (26.35)	
90–99	401 (23.23)	1,762 (19.54)		68 (21.05)	240 (21.22)	
^3^100	133 (7.71)	1,117 (12.39)		28 (8.67)	164 (14.50)	
Residence			<0.001			<0.001
City	341 (19.76)	2,247 (24.92)		35 (10.84)	246 (21.75)	
Town	580 (33.60)	2,915 (32.33)		124 (38.39)	398 (35.19)	
Rural	805 (46.64)	3,855 (42.75)		164 (50.77)	487 (43.06)	
Sex			<0.001			0.055
Female	1,046 (60.60)	4,555 (50.52)		221 (68.42)	708 (62.60)	
Male	680 (39.40)	4,462 (49.48)		102 (31.58)	423 (37.40)	
BMI (kg/m^2^)			0.274			0.137
18.5–23.9	883 (51.16)	4,463 (49.50)		165 (51.08)	527 (46.60)	
<18.5	239 (13.85)	1,205 (13.36)		67 (20.74)	209 (18.48)	
24.0–27.9	400 (23.17)	2,257 (25.03)		51 (15.79)	233 (20.60)	
^3^28.0	156 (9.04)	746 (8.27)		30 (9.29)	82 (7.25)	
Missing	48 (2.78)	346 (3.84)		10 (3.10)	80 (7.07)	
Marital status			<0.001			<0.001
Married and living with a spouse	96 (5.56)	4,800 (53.23)		18 (5.57)	488 (43.15)	
Widowed	1,491 (86.38)	3,922 (43.50)		280 (86.69)	608 (53.76)	
Other	124 (7.18)	201 (2.23)		21 (6.50)	26 (2.30)	
Missing	15 (0.87)	94 (1.04)		4 (1.24)	9 (0.80)	
Education level			<0.001			0.005
0	747 (43.28)	3,198 (35.47)		166 (51.39)	543 (48.01)	
1–6	471 (27.29)	2,752 (30.52)		88 (27.24)	296 (26.17)	
^3^7	233 (13.50)	1,834 (20.34)		23 (7.12)	161 (14.24)	
Missing	275 (15.93)	1,233 (13.67)		46 (14.24)	131 (11.58)	
Self-reported local income status			<0.001			0.017
Poor	180 (10.43)	646 (7.16)		97 (30.03)	260 (22.99)	
General	1,232 (71.38)	6,238 (69.18)		194 (60.06)	733 (64.81)	
Rich	288 (16.69)	2,068 (22.93)		27 (8.36)	129 (11.41)	
Missing	26 (1.51)	65 (0.72)		5 (1.55)	9 (0.80)	
Smoking			0.819			0.224
No	1,419 (82.21)	7,457 (82.70)		286 (88.54)	968 (85.59)	
Yes	287 (16.63)	1,484 (16.46)		34 (10.53)	147 (13.00)	
Missing	20 (1.16)	76 (0.84)		3 (0.93)	16 (1.41)	
Drinking			0.729			0.139
No	1,433 (83.02)	7,451 (82.63)		277 (85.76)	1,000 (88.42)	
Yes	268 (15.53)	1,429 (15.85)		41 (12.69)	111 (9.81)	
Missing	25 (1.45)	137 (1.52)		5 (1.55)	20 (1.77)	
Exercise			0.267			0.609
No	1,105 (64.02)	5,674 (62.93)		238 (73.68)	854 (75.51)	
Yes	592 (34.30)	3,233 (35.85)		77 (23.84)	256 (22.63)	
Missing	29 (1.68)	110 (1.22)		8 (2.48)	21 (1.86)	
Self-reported sleep status			0.029			0.064
General	558 (32.33)	2,779 (30.82)		127 (39.32)	437 (38.64)	
Bad	219 (12.69)	995 (11.03)		148 (45.82)	463 (40.94)	
Good	949 (54.98)	5,243 (58.15)		48 (14.86)	231 (20.42)	
SRH			0.585			0.026
General	664 (38.47)	3,403 (37.74)		157 (48.61)	468 (41.38)	
Bad	157 (9.10)	889 (9.86)		120 (37.15)	439 (38.82)	
Good	905 (52.43)	4,723 (52.38)		46 (14.24)	223 (19.72)	
Missing	0	2 (0.02)		0	1 (0.09)	
Hypertension			0.571			0.603
No	884 (51.22)	4,682 (51.92)		145 (44.89)	528 (46.68)	
Yes	732 (42.41)	3,759 (41.69)		150 (46.44)	510 (45.09)	
Missing	110 (6.37)	576 (6.39)		28 (8.67)	93 (8.22)	
Heart disease			0.740			0.869
No	1,274 (73.81)	6,677 (74.05)		211 (65.33)	751 (66.40)	
Yes	274 (15.87)	1,471 (16.31)		69 (21.36)	252 (22.28)	
Missing	178 (10.31)	869 (9.64)		43 (13.31)	128 (11.32)	
Diabetes			0.075			0.061
No	1,397 (80.94)	7,233 (80.22)		247 (76.47)	836 (73.92)	
Yes	147 (8.52)	899 (9.97)		30 (9.29)	151 (13.35)	
Missing	182 (10.54)	885 (9.81)		46 (14.24)	144 (12.73)	
Stroke			0.080			<0.001
No	1,392 (80.65)	7,183 (79.66)		250 (77.40)	815 (72.06)	
Yes	151 (8.75)	916 (10.16)		24 (7.43)	178 (15.74)	
Missing	183 (10.60)	918 (10.18)		49 (15.17)	138 (12.20)	

### 3.3. Logistic model results

Table 3 reveals the resulting data of the logistic model calculations for depressive symptoms by older adults living alone or not alone in China. Among older adults living alone, age (70–79, OR = 0.352, 95%CI = 0.171–0.722, 80–89:OR = 0.482, 95%CI = 0.240–0.966; 90–99:OR = 0.420, 95%CI = 0.198–0.893), self-reported local income status (average: OR = 0.016; 95%CI = 0.374–0.904), self-reported sleep status (good: OR = 0.262; 95%CI = 0.161–0.425), and SRH (good: OR = 0.297; 95%CI = 0.186–0.472) were noted to act as protective factors. In contrast, self-reported sleep status (bad: OR = 2.613; 95%CI = 1.762–3.874) and SRH (bad: OR = 2.723, 95%CI = 1.781–4.161) were noted to function as risk factors for depressive symptoms. Among older adults living not alone, education level (≥7: OR = 0.639, 95%CI = 0.477–0.856), self-reported local income status (average: OR = 0.406, 95%CI = 0.323–0.511; rich: OR = 0.319, 95%CI = 0.234–0.436), exercise (yes: OR = 0.687-95%CI = 0.563, 0.838), self-reported sleep status (good: OR = 0.396, 95%CI = 0.321–0.489), and SRH (good: OR = 0.524, 95%CI = 0.423–0.650) were protective factors, and marital status (widowed: OR = 1.382, 95%CI = 1.109–1.720), self-reported sleep status (bad: OR = 2.462, 95%CI = 2.015–3.010), SRH (bad, OR = 2.557, 95%CI = 2.080–3.143), and diabetes (yes: OR = 1.304, 95%CI = 1.011–1.682) were risk factors for depressive symptoms.

**Table 3:** Results of the logistic model in older adults living alone and not alone in China.

Variables	Alone			Not alone		
	OR	95%CI	*P*	OR	95%CI	*P*
Age (years)						
<70	Reference			Reference		
70–79	0.352	0.171, 0.722	0.004	1.134	0.856, 1.504	0.380
80–89	0.482	0.240, 0.966	0.040	0.919	0.675, 1.253	0.594
90–99	0.420	0.198, 0.893	0.024	0.884	0.622, 1.257	0.492
^3^100	0.441	0.177, 1.098	0.079	0.889	0.600, 1.319	0.559
Residence						
City	Reference			Reference		
Town	1.443	0.803, 2.593	0.220	0.908	0.706, 1.168	0.451
Rural	1.488	0.835, 2.653	0.177	0.802	0.626, 1.029	0.083
Sex						
Female	Reference			Reference		
Male	1.095	0.706, 1.699	0.686	0.968	0.788, 1.190	0.758
BMI (kg/m^2^)						
18.5–23.9	Reference			Reference		
<18.5	1.335	0.829, 2.150	0.234	1.085	0.853, 1.381	0.505
24.0–27.9	0.729	0.453, 1.173	0.192	1.002	0.809, 1.241	0.985
^3^28.0	0.976	0.523, 1.824	0.940	1.048	0.763, 1.439	0.774
Marital status						
Married and living with a spouse	Reference			Reference		
Widowed	0.777	0.374, 1.615	0.500	1.382	1.109, 1.720	0.004
Other	0.601	0.218, 1.651	0.323	1.084	0.596, 1.973	0.791
Education level						
0	Reference			Reference		
1–6	0.919	0.606, 1.393	0.690	0.814	0.658, 1.006	0.056
^3^7	0.771	0.394, 1.508	0.447	0.639	0.477, 0.856	0.003
Self-reported local income status						
Poor	Reference			Reference		
General	0.582	0.374, 0.904	0.016	0.406	0.323, 0.511	<0.001
Rich	0.632	0.323, 1.237	0.181	0.319	0.234, 0.436	<0.001
Smoking						
No	Reference			Reference		
Yes	0.761	0.430, 1.349	0.350	0.943	0.718, 1.239	0.674
Drinking						
No	Reference			Reference		
Yes	0.900	0.517, 1.568	0.710	0.916	0.694, 1.210	0.536
Exercise						
No	Reference			Reference		
Yes	0.864	0.577, 1.294	0.478	0.687	0.563, 0.838	<0.001
Self-reported sleep status						
General	Reference			Reference		
Bad	2.613	1.762, 3.874	<0.001	2.462	2.015, 3.010	<0.001
Good	0.262	0.161, 0.425	<0.001	0.396	0.321, 0.489	<0.001
SRH						
General	Reference			Reference		
Bad	2.723	1.781, 4.161	<0.001	2.557	2.080, 3.143	<0.001
Good	0.297	0.186, 0.472	<0.001	0.524	0.423, 0.650	<0.001
Hypertension						
No	Reference			Reference		
Yes	1.311	0.909, 1.890	0.147	1.023	0.853, 1.227	0.806
Heart disease						
No	Reference			Reference		
Yes	1.046	0.664, 1.650	0.846	0.836	0.668, 1.045	0.115
Diabetes						
No	Reference			Reference		
Yes	0.960	0.523, 1.762	0.895	1.304	1.011, 1.682	0.041
Stroke						
No	Reference			Reference		
Yes	0.597	0.314, 1.135	0.116	1.190	0.927, 1.526	0.172

Hence, the differences in depressive symptoms observed between individuals living alone and those in not-alone arrangements in China can be attributed to 3 primary areas. First, age (70–79: OR = 0.352, 95%CI = 0.171–0.722; 80–89:OR = 0.482, 95%CI = 0.240–0.966; 90–99:OR = 0.420, 95%CI = 0.198–0.893) was a protective factor only in older adults living alone. Second, education level (≥7: OR = 0.639, 95%CI = 0.477–0.856), self-reported local income status (rich: OR = 0.319, 95%CI = 0.234–0.436), and (yes: OR = 0.687-95%CI = 0.563, 0.838) were observed to be protective factors only in older adults living not alone. Third, marital status (widowed, OR = 1.382) and diabetes (yes, OR = 1.304) were risk factors only in older adults living not alone.

### 3.4. Fairlie decomposition analysis results

Table 4 depicts the resulting data of the decomposition model of the variation in depressive symptoms across older adults living alone and not alone in China. The results showed that 55.33% of the difference in depressive symptoms was owing to observed factors, whereas 44.67% was owing to factors involving living alone and not alone and other unobserved factors. To ensure the robustness Fairlie model, a multiple-impute model was established for supplementary analysis (supplement Table S1). Among the observed factors, specific elements played a remarkable role in explaining the differences in depressive symptoms (*p* < 0.05). These factors included marital status (20.55%), education level (4.63%), self-reported local income status (7.25%), self-reported sleep status (17.56%), and SRH (4.24%).

**Table 4:** Fairlie decomposition of depressive symptoms disparity between older adults living alone and not alone in China.

Terms of decomposition	DS	
Difference	0.04563494	
Explained (%)	0.02524908 (55.33)	
Non-explained (%)	0.02038586 (44.67)	
Explained			
Contribution to difference	*β*	Contribution (%)	(95%CI)
Age	−0.0006219	−1.36	−0.0017896, 0.0005458
Residence	−0.0005276	−1.16	−0.0019317, 0.0008765
Sex	0.0010026	2.20	−0.0001522, 0.0021575
BMI	0.0000908	0.20	−0.0003855, 0.0005671
Marital status	0.0093795	20.55	0.0022295, 0.0165295
Education level	0.0021131	4.63	0.0005509, 0.0036753
Self-reported local income status	0.0033106	7.25	0.0020159, 0.0046053
Smoking	0.0001247	0.27	−0.0003726, 0.000622
Drinking	0.0000453	0.10	−0.0002045, 0.0002951
Self-reported sleep status	0.0080152	17.56	0.0065413, 0.0094892
Exercise	−0.0001395	−0.31	−0.0005734, 0.0002945
SRH	0.0019342	4.24	0.0011865, 0.002682
Hypertension	0.0003598	0.79	−0.0002153, 0.0009349
Heart disease	0.0003736	0.82	−0.0000976, 0.0008448
Diabetes	−0.0000075	−0.02	−0.0002759, 0.0002607
Stroke	−0.0003119	−0.68	−0.0006278, 0.0000040

## 4. Discussion

This research delved into the relationship between specific factors (e.g., demographic characteristics, sociological characteristics, and health status) and depressive symptoms among older adults living alone and not alone in China. Additionally, the study quantified the degree to which these factors contributed to the discernible variations in depressive symptoms among older adults. This investigation substantiated the presence of remarkable differences in depressive symptoms between older adults living alone and those cohabitating in not-alone arrangements within China.

This study showed that the prevalence of depressive symptoms among Chinese older adults (age ≥ 65) was 11.92%. Notably, this prevalence was considerably lower than that reported in a meta-analysis which indicated a prevalence of approximately 23.6% for depressive symptoms among Chinese older adults aged over 60 (25). The prevalence was recorded to be higher among older adults living alone (15.76%) than among older adults living not alone (11.15%), which was aligned with the data acquired through prior investigation of depressive symptoms among older adults in China (26, 27). This is mainly because living alone makes it easier to experience social isolation, and cognitive decline, which are risk factors for depression according to previous research (28–30). Additionally, the 4.61%-point difference in the prevalence of depressive symptoms between alone and not alone suggested that medical personnel must pay attention to older adults living alone and provide them with more professional support to alleviate any anxiety or depression.

Our logistic regression analysis revealed further differences between the covariates of depressive symptoms in Chinese older adults living alone and not alone. Age, marital status, education level, self-reported local income status, exercise, self-reported sleep status, SRH, and diabetes were associated with the presence of depressive symptoms, similar to the findings of other researchers (6, 31). Specifically, older adults living not alone but widowed had more depressive symptoms because this group lives with their children’s family and had negative interactions with family members because of the generation gap. In China, older adults are often required to care for their grandchildren, which leads to restrictions on social interactions (32). The group living not alone with shorter learning time had more depressive symptoms. This was likely because older adults with higher education time can find more ways to release their emotions and seek more ways like the Internet to treat depression symptoms, which was inconsistent with previous studies (33). Poor self-reported local income status would increase the risk of depressive symptoms, which can be attributed to the fact that older adults with lower income have lower life quality and limited access to treatment for depressive symptoms and other mental health issues (32, 34). The results indicated that lack of sleep was associated with more depressive symptoms in older adults, this was associated with cognitive decline which was confirmed to be prone to depression (35). SRH was based on subjective perceptions of their health, so Poor SRH means they were more dissatisfied with their health and found it difficult to actively participate in their lives (36). Additionally, incongruent with other findings (11, 37), the older adults living alone in this study were less likely to have depressive symptoms; however, the specific reasons for this require further investigation.

The results of the Fairlie model showed that this part of the difference was related to marital status (20.55%), education level (4.63%), self-reported local income status (7.25%), self-reported sleep status (17.56%), and SRH (4.24%). All factors were intervenable. If these intervening factors could be improved, the difference in depressive symptoms between Chinese older adults living alone and not alone could be reduced by about 55.33%. Thus, our study can provide targeted measures to reduce depression in older adults living alone according to the results of the Fairlie model. First, we should help older adults develop healthy lifestyles (i.e., regular exercise, and dietary patterns) while ensuring adequate sleep time and participation in social activities. Second, we should enhance protection for older adults living in poverty, especially in terms of health and other relevant aspects. Third, the government should pay attention to targeted older adults in need and encourage more young people to pay attention to the health of the targeted older adults through publicity, especially younger older adults who live alone, older adults who do not live alone but who have been widowed, and less educated older adults who do not live alone and in poverty. They should be offered an appropriate tilt in health insurance policies, and targeted assistance and aid programs should be formulated for them.

### 4.1. Limitations

This research was limited in certain respects. First, the definition of depressive symptoms was based on the CES-D-10 scale, which, although widely validated and boasting good reliability, remains reliant on self-reporting, thus introducing potential inaccuracies in contrast to medical diagnoses. Second, the domain of factors influencing depressive symptoms is multifaceted, and while this research encompassed a subset of indicators, other significant variables (such as polypharmacy, dementia, osteoporosis, osteoarthritis, etc.) might not have been accounted for. Last, the expansive older adult population of China poses a challenge, as the CLHLS dataset utilized in this study covers only a fraction of this demographic and therefore cannot fully cover the entirety of older adults within the country.

## 5. Conclusion

The study findings contribute novel insights into the distinctions between older adults living alone and those living not alone within China. These results are poised to play a pivotal role in the refinement and establishment of mental health prevention and treatment policies targeted toward older adults in China. By accurately identifying the factors that influence living alone versus not alone conditions and their varying impacts on depressive symptoms, the groundwork is laid for the development of targeted and precise intervention strategies, aimed at enhancing the mental well-being of high-risk segments of the older adults. Ultimately, the problem of living alone and not alone differences in depressive symptoms will be effectively addressed.

## Data availability statement

The datasets presented in this study can be found in online repositories. The names of the repository/repositories and accession number(s) can be found in the article/Supplementary material.

## Ethics statement

The data for this study were taken from the CLHLS, which is organized by the Center for Healthy Aging and Development Studies at Peking University and has been approved by the Research Ethics Committees of Peking University and Duke University. The data analyzed here are available in the public domain, and therefore, separate ethical approval was not required for this study.

## Author contributions

CH: Formal Analysis, Funding acquisition, Project administration, Writing – original draft. ZD: Conceptualization, Data curation, Formal Analysis, Writing – original draft. HL: Conceptualization, Methodology, Project administration, Supervision, Validation, Writing – original draft. SL: Conceptualization, Methodology, Project administration, Writing – review & editing. MD: Data curation, Investigation, Project administration, Writing – review & editing. TL: Conceptualization, Investigation, Methodology, Project administration, Writing – review & editing. LY: Data curation, Project administration, Supervision, Writing – review & editing.

## Funding

The author(s) declare financial support was received for the research, authorship, and/or publication of this article. This work was sponsored by the Shanghai Sailing Program, Prediction of psychological stress of urban major infectious disease groups based on multi-agent modeling, 21YF1457500.

## Conflict of interest

The authors declare that the research was conducted in the absence of any commercial or financial relationships that could be construed as a potential conflict of interest.

## Publisher’s note

All claims expressed in this article are solely those of the authors and do not necessarily represent those of their affiliated organizations, or those of the publisher, the editors and the reviewers. Any product that may be evaluated in this article, or claim that may be made by its manufacturer, is not guaranteed or endorsed by the publisher.
